# A Well Deserved Honor

**Published:** 1895-08

**Authors:** 


### EDITORIAL.

### A Well Deserved Honor.

We have just learned that Dr. L. P. Bethel, of Kent, Ohio, editor of the *Ohio Dental Journal*, has been elected to a full Professorship, in the Dental Department of the Western Reserve University.

We extend congratulations. The institution has honored itself in this selection. Dr. Bethel is well qualified for any work he will undertake.

